# Plasma lipidomics of primary biliary cholangitis and its comparison with Sjögren’s syndrome

**DOI:** 10.3389/fimmu.2023.1124443

**Published:** 2023-05-05

**Authors:** Haolong Li, Haoting Zhan, Linlin Cheng, Yuan Huang, Xiaomeng Li, Songxin Yan, Yongmei Liu, Li Wang, Yongzhe Li

**Affiliations:** ^1^ Department of Clinical Laboratory, State Key Laboratory of Complex Severe and Rare Diseases, Peking Union Medical College Hospital, Chinese Academy of Medical Science and Peking Union Medical College, Beijing, China; ^2^ Department of Rheumatology, National Clinical Research Center for Dermatologic and Immunologic Diseases (NCRC-DID), Peking Union Medical College Hospital, Chinese Academy of Medical Science and Peking Union Medical College, Beijing, China

**Keywords:** lipidomics, primary biliary cholangitis, Sjögren’s syndrome, plasma, ursodeoxycholic acid

## Abstract

**Background:**

Abnormal lipid metabolism is common in patients with primary biliary cholangitis (PBC). PBC and Sjögren’s syndrome (SS) frequently coexist in clinical practice; however, the lipid characteristics of both diseases are unknown. Therefore, we aimed to analyze the plasma lipid profiles of both diseases.

**Methods:**

Plasma samples from 60 PBC patients, 30 SS patients, and 30 healthy controls (HC) were collected, and untargeted lipidomics was performed using ultrahigh-performance liquid chromatography high-resolution mass spectrometry. Potential lipid biomarkers were screened through an orthogonal projection to latent structure discriminant analysis and further evaluated using receiver operating characteristic (ROC) analysis.

**Results:**

A total of 115 lipids were differentially upregulated in PBC patients compared with HC. Seventeen lipids were positively associated with the disease activity of PBC, and ROC analysis showed that all of these lipids could differentiate between ursodeoxycholic acid (UDCA) responders and UDCA non-responders. The top six lipids based on the area under the curve (AUC) values were glycerophosphocholine (PC) (16:0/16:0), PC (18:1/18:1), PC (42:2), PC (16:0/18:1), PC (17:1/14:0), and PC (15:0/18:1). In comparison with SS, 44 lipids were found to be differentially upregulated in PBC. Additionally, eight lipids were found to have a good diagnostic performance of PBC because of the AUC values of more than 0.9 when identified from SS and HC groups, which were lysophosphatidylcholines (LysoPC) (16:1), PC (16:0/16:0), PC (16:0/16:1), PC (16:1/20:4), PC (18:0/20:3), PC (18:1/20:2), PC (20:0/22:5), and PC (20:1/22:5).

**Conclusion:**

Our study revealed differentially expressed lipid signatures in PBC compared with HC and SS. PC is the main lipid species associated with disease activity and the UDCA response in patients with PBC.

## Introduction

Primary biliary cholangitis (PBC) is an autoimmune cholestatic liver disease characterized by the progressive destruction of intrahepatic bile duct epithelial cells (1). PBC mainly occurs in middle-aged women over 40 years old, with an estimated global prevalence of 146 per million (2). Most patients are asymptomatic in the early stage of PBC, whereas some commonly present with fatigue and pruritus (3). Regarding laboratory tests, patients with PBC usually have increased serum alkaline phosphatase (ALP) and glutamyl transpeptidase (GGT). Meanwhile, approximately 90% of PBC patients are positive for anti-mitochondrial antibody (AMA), particularly the AMA-M2 subtype, a serological hallmark of PBC diagnosis (4). Patients with negative AMA or AMA-M2 require invasive assessment, such as liver biopsy, to confirm the diagnosis. Ursodeoxycholic acid (UDCA) is the first-line drug for PBC therapy, but nearly 40% of PBC patients do not respond sufficiently to it (5). Patients with an incomplete response to UDCA have a shorter survival and a higher risk of developing liver cirrhosis and liver failure than those who respond (6). In contrast, biomarkers for discriminating between two types of PBC patients with significantly different prognoses are rare.

PBC is susceptible to complications from other autoimmune diseases; more than 60% of patients with PBC have other autoimmune diseases, including Sjögren’s syndrome (SS), autoimmune thyroid disease, rheumatoid arthritis, and systemic sclerosis (7). With a prevalence of 35% in PBC (8), SS is one of the most common autoimmune extrahepatic conditions; it is characterized by the destruction of exocrine glands, with typical symptoms of dry eyes and dry mouth. PBC and SS have many common features, including female predisposition, fatigue, and pruritus (9). They are also regarded as autoimmune epithelitis because epithelial cell apoptosis is critical in these two diseases (9). Many studies have revealed the differences and similarities in genetics and immunology between PBC and SS (10), but few studies have concentrated on comparing the metabolic characteristics of both diseases.

Lipidomics, a high-throughput method for analyzing the function and structure of lipids in various samples, has been extensively used in exploring pathophysiology. Lipids play vital roles in many biological functions, including energy storage, signal transmission, and inflammatory responses. Lipid abnormalities are commonly observed in PBC because of cholestasis, and the most significant change in serum lipids in PBC is low-density lipoprotein cholesterol caused by elevated LP-X (11). In addition, PBC is also closely associated with bile acid metabolism abnormalities; thus, previous studies have focused on measuring the metabolite contents in serum and urine from patients with PBC by metabolomics to explore non-invasive biomarkers for PBC (12, 13). However, no study has investigated lipid profiles via lipidomic technology in the plasma of patients with PBC.

In this study, we used untargeted lipidomics to identify a distinctive lipid signature in plasma samples from patients with PBC and discover lipid biomarkers that could reflect the UDCA response in patients with PBC. Moreover, we compared the lipid profiles of patients with PBC and SS and explored potential lipid markers for PBC diagnosis.

## Materials and methods

### Subjects and sample collection

Sixty PBC patients and 30 healthy controls (HC) matched for age and sex were recruited from Peking Union Medical College Hospital (PUMCH), respectively. The diagnosis of PBC was based on generally accepted criteria, according to the practice guidelines of the European Association for the Study of Liver Diseases for PBC. PBC patients who were untreated or who only received UDCA treatment with 13–15 mg/kg/day for greater than 12 months were included (14). HC were collected from health check-up population with normal physical examination results and no self-reported diseases and did not take any medication for the last 6 months. In addition, 30 untreated SS patients fulfilled the 2002 American European Consensus Group criteria (15) and were also enrolled. Patients accompanied by other autoimmune diseases, metabolic disease, and malignant diseases were not enrolled in this study. All the participants were Asian and did not take any lipid-lowering drugs. Detailed clinical characteristics of the participants are summarized in **Table 1**. This study was reviewed and approved by the Institutional Review Board of PUMCH (JS-2156). All subjects provided written informed consent. Overall, 40 patients with PBC received UDCA therapy with 13–15 mg/kg/day for more than 1 year. Furthermore, we categorized these patients into two groups: UDCA responder (*n* = 20) and UDCA non-responder (*n* = 20), which were established using the POISE criteria (16) [ALP <1.67 × ULN (upper limit of normal value) and bilirubin <1 × ULN)]. Patients who did not meet the POISE criteria were graded as UDCA non-responders, whereas the others were regarded as UDCA responders. Additionally, 20 patients with PBC were newly diagnosed without any treatment.

**Table 1:** Demographic and laboratory features of healthy controls, primary biliary cholangitis patients, and Sjögren’s syndrome patients.

Group	HC (*n* = 30)	PBC (*n* = 60)	SS (*n* = 30)	*p*-values		
				PBC vs. HC	SS vs. HC	PBC vs. SS
Age, years	52.73 ± 11.08	53.13 ± 11.82	46.10 ± 11.87	1	0.086	0.023
Female sex	26 (86.7%)	54 (90.0%)	29 (96.7%)	0.635	0.161	0.266
Smoking history, *n* (%)	0	1 (1.7%)	0	0.477	NA	0.477
Drinking history, *n* (%)	0	1 (1.7%)	0	0.477	NA	0.477
Serological examinations						
ALP, U/L	70 (50–84)	229 (121–318)	67 (51–81)	<0.001	1	<0.001
GGT, U/L	16 (13–21)	154 (66–298)	18 (13–26)	<0.001	0.473	<0.001
ALT, U/L	15 (12–19)	41 (24–87)	15 (10–22)	<0.001	0.667	<0.001
AST, U/L	19 (16–21)	48 (30–87)	21 (18–24)	<0.001	0.222	<0.001
TP, g/L	70.30 ± 3.84	77.58 ± 6.60	79.97 ± 6.19	<0.001	<0.001	0.224
ALB, g/L	45 (43–46)	43 (40–45)	44 (42–46)	0.012	0.291	0.197
TBA, µmol/L	1.45 (0.88–2.15)	11.95 (4.98–25.58)	2.10 (1.18–3.85)	<0.001	0.169	<0.001
TBIL, µmol/L	12.30 (9.75–14.98)	14.65 (11.35–20.13)	8.85 (7.75–12.00)	0.031	<0.001	<0.001
DBIL, µmol/L	3.55 (2.78–4.55)	5.10 (3.13–8.55)	3.10 (2.38–3.63)	0.005	0.288	<0.001
IgG, g/L	ND	14.31 (12.69–17.37)	18.94 (14.89–24.77)	NA	NA	<0.001
IgA, g/L	ND	2.62 (1.80–3.36)	2.85 (2.02–3.59)	NA	NA	0.392
IgM, g/L	ND	2.78 (1.92–4.32)	1.09 (0.89–1.59)	NA	NA	<0.001
C3	ND	ND	1.01 ± 0.16	NA	NA	NA
C4	ND	ND	0.17 ± 0.06	NA	NA	NA
ESR	ND	ND	25 (10–32)	NA	NA	NA
RF	ND	ND	103 (44–147)	NA	NA	NA
CRP	ND	ND	0.70 (0.40–1.00)	NA	NA	NA
ANA, %	ND	58 (96.7%)	29 (96.7%)	NA	NA	1
AMA, %	ND	48 (80.0%)	ND	NA	NA	NA
AMA, M2%	ND	47 (78.3%)	ND	NA	NA	NA
Anti-GP210, %	ND	15 (28.8%) ^a^	ND	NA	NA	NA
Anti-SP100, %	ND	14 (26.9%) ^a^	ND	NA	NA	NA
Anti-SSA, %	ND	ND	27 (90.0%)	NA	NA	NA
Anti-SSB, %	ND	ND	16 (53.3%)	NA	NA	NA
Anti-Ro52, %	ND	ND	25 (83.3%)	NA	NA	NA
Medication						
UDCA 13–15 mg/kg/day, *n* (%)	NA	40 (66.7%)	NA	NA	NA	NA

Values were presented as mean ± SD, median (interquartile range), or number (percentage).

ALB, albumin; ALP, alkaline phosphatase; ALT, alanine aminotransferase; AMA, antimitochondrial antibody; ANA, antinuclear antibody; AST, aspartate transaminase; BUN, blood urea nitrogen; Cr, creatinine; DBIL, direct bilirubin; GGT, gamma glutamyl transferase; GP210, glycoprotein-210; HDL, high-density lipoprotein; IgA, immunoglobulin A; IgG, immunoglobulin G; IgM, immunoglobulin M; LDL, low-density lipoprotein; NA, not available; ND, not detected; TBA, total bile acid; TBIL, total bilirubin; TC, total cholesterol; TG, total triglycerides; TP, total protein; UA, uric acid; UDCA, ursodeoxycholic acid.

a Available in 52 patients with PBC.

All individuals fasted overnight before blood sampling. Whole blood was collected from each subject in ethylenediaminetetraacetic acid-treated tubes. Plasma was separated by centrifugation at 1,000 × *g* for 10 min and stored at −80°C.

### Sample preparation

Lipids extracted from plasma were processed through the following steps: (1) Frozen plasma samples were thawed at room temperature, and then 20 μl of plasma was pipetted to a 2-ml EP tube; (2) 120 μl of methanol was added to each of the plasma samples, followed by 3 min of vortex oscillation; (3) 360 μl of methyl tert-butyl ether (Sigma Aldrich Co., St. Louis, MO, USA) and 100 μl of ultrapure water were added and then vortexed for 10 min; (4) the samples were put on ice for 10 min before centrifugation at 13,000 rpm at 4°C for 15 min; (5) 200 μl of supernatant was collected from each sample separately pipetted into the new 1.5-ml EP tube, vacuum-dried, and then stored at −80°C for 1 h; (6) the dried lipid extract was dissolved with 80 μl of reconstitution reagent (65% acetonitrile (ACN):30% iso-propyl alcohol (IPA):5% H_2_O, v:v:v); and (7) 70 μl of the aforementioned dissolved sample from each tube was then transferred into vials for Liquid Chromatograph-Mass Spectrometer (LC-MS) analysis.

To evaluate the accuracy of the lipid detected by the instrument, an extra 200 μl of supernatant in step (5) was collected from each sample and then mixed for pooled quality control (QC). The pooled QC samples were then dried under a vacuum and subjected to the same procedure as the other plasma samples.

### UPLC-HRMS analysis

As previously reported, we adopted analytical methods in this study, except for column changes (17). Lipidomics analysis was performed using a Q Exactive Plus high-resolution mass spectrometer (Thermo Scientific, USA) equipped with an Ultimate 3000 UHPLC system (Thermo Scientific, USA). Chromatographic separation conditions were maintained for untargeted lipidomic analysis of the hydrophobic fraction for both positive and negative ionization detection methods. An Accucore C30 core-shell column (Thermo Scientific, San Jose, CA, USA, 2.6 μm, 2.1 × 100 mm) was used for lipid separation at 50°C, which was eluted with 60% acetonitrile in water (A) and 10% acetonitrile in isopropanol (B), both containing 10 mM ammonium formate and 0.1% formic acid. The separation gradient was optimized as follows: initial 10% B ramping to 50% in 5 min, further increasing to 100% in 23 min, and another 7 min for column washing and equilibration using 0.3 ml/min flow rate.

For lipid detection, the quadrupole-Orbitrap mass spectrometer was operated under identical ionization parameters with a heated electrospray ionization source except for ionization voltage (positive 3.5 kV, negative 3.0 kV) including sheath gas 45 arb, aux gas 10 arb, heater temperature 355°C, capillary temperature 320°C, and S-Lens RF level 55%. The lipidome extracts were profiled from 300 to 2,000 m/z in the full scan mode at 70,000 full width at half maxima (FWHM) resolution with automatic gain control (AGC) 1E6 and 200 ms maximum injection time. Lipid molecules were structurally identified by acquiring data-dependent MS (2) spectra under both positive and negative ionization, including 70,000 FWHM full scan resolution, 17,500 FWHM MS/MS resolution, loop count 10, AGC target 3E6, and maximum injection time 200 ms and 80 ms for full scan and MS/MS, respectively. Dynamic exclusion 8 s and stepped normalized collision energy 25% + 40% and 35% were employed for positive and negative modes, respectively, after optimization.

### Data pre-processing

The acquired untargeted lipidomics data were processed using Lipid Search software, including peak picking and lipid identification. The acquired MS (2) spectra were searched against *in silico* predicted spectra of diverse lipids. The mass accuracy for precursor and MS/MS product ion searching were 5 ppm and 5 mDa, respectively. The MS/MS similarity score threshold was set at 5. The potential ionization adducts include hydrogen, sodium, and ammonium for the positive mode, and hydrogen, formate, and acetate for the negative mode. Lipid identification was strictly manually checked and investigated individually to eliminate false positives chiefly based on peak shake, adduct ion behavior, fragmentation pattern, and chromatographic behavior. The chemical identification results were finally annotated using the classification criteria proposed by MSI (lipidomics standardization initiative) (18).

### Statistical analysis

The lipidomic data from different measurements were merged, and missing values were refilled with a constant of 10,000, if they had not been detected in certain samples after manual checking. Finally, Log2 was transformed for the final statistical analysis. Student’s *t*-test was used for comparison between two groups and logistic regression analysis was used to adjust for the age when comparing between PBC and SS groups. False discovery rate (FDR) was adjusted using the Benjamini–Hochberg method. Differences between more than two groups were analyzed using a one-way analysis of variance followed by Bonferroni post-hoc tests or Kolmogorov–Smirnov test followed by Dunn’s test. For multivariate analyses, principal component analysis (PCA) and orthogonal partial least squares discriminant analysis (OPLS-DA) were used to evaluate the ability of the lipids to distinguish cases from controls. Pathway enrichment analysis was performed on Lipid Pathway Enrichment Analysis (LIPEA) using the Kyoto Encyclopedia of Genes and Genomes (KEGG) database. A receiver operating characteristic (ROC) curve was used to validate the accuracy of all annotated differential lipids using the R package ROCR. Based on profiling, unsupervised consensus clustering was used to classify PBC lipid subtypes and analyzed using the R package ConsensusClusterPlus with differential lipids (*n* = 115) between HC and PBC. The following detail settings were used for clustering: number of repetitions = 50 bootstraps; PItem = 0.8 (resampling 80% of any sample); pFeature = 1 (resampling 100% of any lipid). The cluster number varied from 2 to 6, and the optimal cluster number that produced the most stable consensus matrices and the most unambiguous cluster assignments across permuted clustering runs was selected. The number of clustering was based on the clearest cut between varied clusters (19) and the delta plot of the relative change in the area under the cumulative distribution function (CDF) curve (20). The final clusters identified as such correspond to lipid subtypes of PBC. Differential lipids were defined with an FDR < 0.05 and fold change > 2, or < 0.5. Furthermore, differential lipids with variable importance in projection (VIP) > 1 were used for biomarker analysis. Data were analyzed using IBM SPSS version 23.0 software (IBM Corp., Armonk, NY, USA) and MetaboAnalyst 5.0 (21). Hiplot (https://hiplot.org) (22) was used to visualize the data.

## Results

### Substantial changes in the lipid profile of patients with PBC

Untargeted lipidomics of 120 subjects (30 HC, 60 PBC, and 30 SS) was performed using a high-resolution LC-MS/MS platform. The PCA score plot showed that five QC samples were located close to each other (**Figure S1A**). In addition, it was clear from the relative standard deviation distribution that among the 480 lipids identified, 97.49% had relative standard deviations < 30% (**Figure S1B**). We further annotated the distribution of 480 identified lipids, showing that untargeted lipidomics provides high coverage of the main lipid species in the plasma of our sample. Therefore, the instrument analysis system for lipidomics detection and the test data from our experiment were stable and reliable.

**Figure 1:**
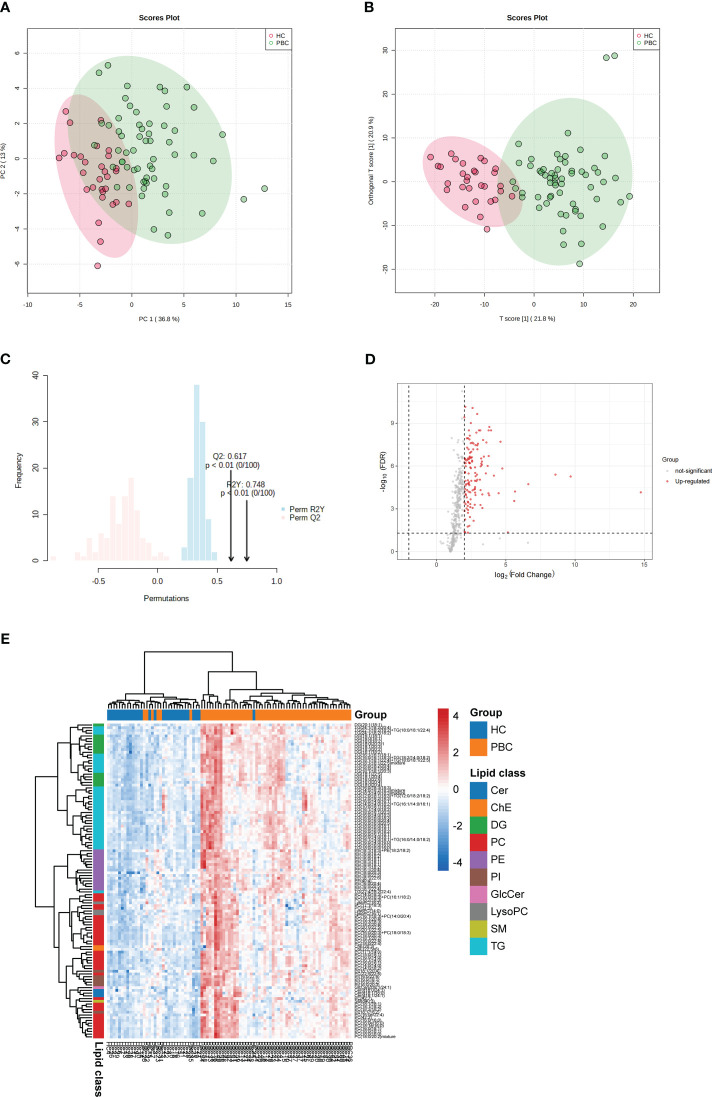
Identification of lipid profiling in plasma of PBC. **(A)** Unsupervised PCA model for all PBC and HC groups. **(B)** Supervised OPLS-DA model shows discrimination between the PBC and HC groups. **(C)** Permutation test for the OPLS-DA model. **(D)** Volcano map of differentially expressed lipids between PBC and HC groups. Red represents the upregulated lipid and non-significant lipid molecules are gray; differential metabolic features were selected by the following criteria: FDR < 0.05 and fold change > 2.0 or < 0.5. **(E)** Clustering heatmap of the 115 lipids that were differentially expressed and VIP > 1 illustrated by the OPLS-DA model between PBC and HC groups. Cer, ceramide; ChE, cholesterol and cholesterol ester; DG, diacylglycerol; PC glycerophosphocholine; PE, glycerophosphoethanolamine; PI, glycerophosphoinositol; GlcCer, glycosphingolipid; LysoPC, lysophosphatidylcholines; SM, sphingomyelin; TG, triacylglycerol.

To explore the differences in lipid levels between patients with PBC and HC, PCA and OPLS-DA were performed. In the PCA model, the proportion of the variance explained by PC1 and PC2 was 49.8%. There was no clear separation between the PBC and HC groups in the PCA model (**Figure 1A**). To further differentiate between the PBC and HC groups, OPLS-DA was performed. In the OPLS-DA model, a clear difference between the lipid profiles of the PBC and HC groups was observed (**Figure 1B**), with *R* ^2^ *Y* (cum) = 0.471 and *Q* ^2^ (cum) = 0.444. Overfitting was validated by permutation testing to the OPLS-DA model, which showed that a *p*-value < 0.01 is at 100 permutations (**Figure 1C**). The above data indicated good predictive performance, and this model was not overfitting. A total of 132 differential lipids were upregulated, and no differential lipids were downregulated in the PBC group compared to the HC group, as indicated by the volcano plot (**Figure 1D**; **Table S1**). The OPLS-DA model, which was used for clustering and is shown in the heatmap annotated with lipid species, shows that 115 of 132 differential lipids had VIP > 1, as illustrated by the clustering (**Figure 1E**). A clear distinction was observed between PBC and HC samples with HC having higher enrichment for most lipid species, including ceramide (Cer), cholesterol and cholesterol ester (ChE), diacylglycerol (DG), glycerophosphocholine (PC), glycerophosphoethanolamine (PE), glycerophosphoinositol (PI), glycosphingolipid (GlcCer), lysophosphatidylcholines (LysoPC), sphingomyelin (SM), and triacylglycerol (TG).

### Pathway analysis of differential lipids in PBC and HC

**Figure 2:**
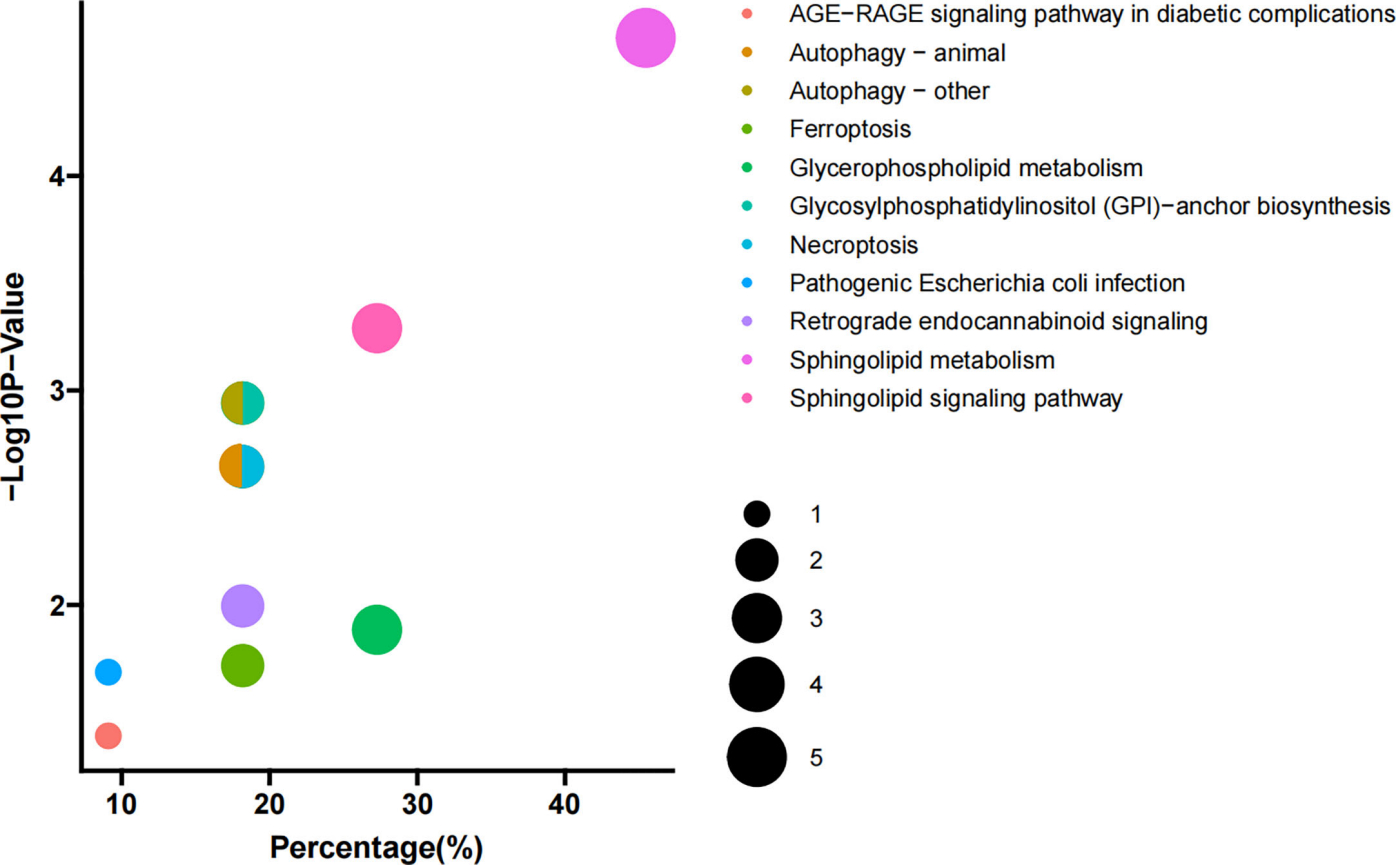
Pathway enrichment. Enriched metabolic pathways involving the differential lipids between PBC and HC groups. The sizes of the dots indicate number of lipids in each pathway; the *x*-axis indicates the number of lipids in one pathway over total number of enriched lipids, converted to percentage; the *y*-axis represents the −Log10 *p*-value.

KEGG pathway analysis was used to determine the lipid metabolomic pathways affected in patients with PBC. Eleven lipid metabolomic pathways were selected and evaluated (**Figure 2**). The differential lipids were enriched in the following nine pathways that were considered of the greatest significance [*p* < 0.05, converted lipids (percentage) > 15%]: sphingolipid metabolism, sphingolipid signaling pathway, glycosylphosphatidylinositol (GPI)–anchor biosynthesis, autophagy—other, autophagy—animals, necroptosis, retrograde endocannabinoid signaling, glycerophospholipid metabolism, and ferroptosis. The detailed results of these pathways are presented in **Table S2**.

### Lipids that showed association with the disease activity of PBC

**Figure 3:**
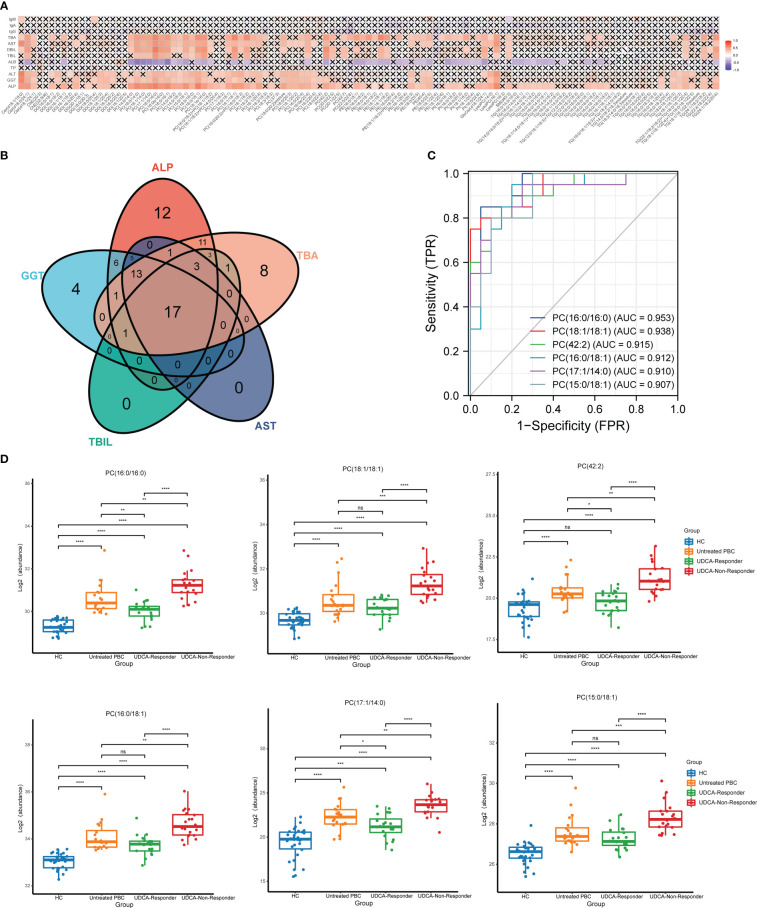
The lipids that could reflect the disease activity of PBC. **(A)** Correlation analysis of key laboratory indicators and the significantly changed lipids. **(B)** The Venn plot shows that 17 lipids simultaneously correlated with the ALP, GGT, TBIL, AST, and TBA. **(C)** The ROC analysis indicated that the top six lipids could identify UDCA responder and UDCA non-responder based on AUC. **(D)** The top six lipids expressed in HC, untreated PBC, UDCA responder, and UDCA non-responder groups. ns, not significant. *p < 0.05, **p < 0.01, ***p < 0.001, ****p < 0.0001.

The correlations between the relative levels of 115 differential lipids in the plasma of patients with PBC are shown in **Figure S2**. Most lipids showed a significantly positive correlation with various lipid species, and the strongest correlations were between PC and PI. In addition, we examined the correlation between 115 differential lipids and laboratory examinations, including ALP, GGT, total bilirubin (TBIL), aspartate transaminase (AST), total bile acid (TBA), total protein (TP), albumin (ALB), direct bilirubin (DBIL), alanine aminotransferase (ALT), immunoglobulin G (IgG), immunoglobulin A (IgA), and immunoglobulin M (IgM) (**Figure 3A**). The detailed correlation coefficients for each lipid are presented in **Table S3**. The data showed that 74 lipids were positively associated with ALP, 48 lipids were positively associated with ALT, 47 lipids were positively associated with GGT, 59 lipids were positively associated with TBA, 39 lipids were positively associated with TBIL, 25 lipids were positively associated with TBIL, 4 lipids were positively associated with DBIL, and 2 lipids were positively associated with IgM. In addition, 49 lipids were negatively associated with ALB, whereas none were correlated with TP, IgG, and IgM.

To explore the lipids that could be biomarkers that reflect the disease activity of PBC, we selected lipids that simultaneously correlated with ALP, GGT, TBIL, AST, and TBA. We observed that 17 lipids simultaneously correlated with ALP, GGT, TBIL, AST, and TBA (**Figure 3B**). Thirteen lipids were PC, two lipids were TG, and the others were LysoPC and Cer. Moreover, we classified the patients with PBC into three groups: untreated PBC, UDCA responders, and UDCA non-responders. The laboratory features of the three groups of patients with PBC are shown in **Table S4**. ROC analysis was used to validate the accuracy of lipids in distinguishing between the UDCA responders and UDCA non-responders. The results showed that all 17 lipids had moderate identification ability, and the AUC values of 17 lipids were all above 0.7 (**Table 2**). The top six lipids based on AUC values were PC (16:0/16:0), PC (18:1/18:1), PC (42:2), PC (16:0/18:1), PC (17:1/14:0), and PC (15:0/18:1), and the AUC values of the top six lipids were all above 0.9 (**Figure 3C**). All six lipids had the highest level in the UDCA non-responder group, which was significantly higher than that in the HC, untreated PBC, and UDCA responder groups (**Figure 3D**). PC (42:2), PC (16:0/16:0), and PC (17:1/14:0) levels in the untreated PBC group were significantly higher than those in the UDCA responder group, whereas no change was observed in the other groups.

**Table 2:** Diagnostic value of the 17 selected lipid metabolites altered in the UDCA responder group compared to the UDCA non-responder group.

Lipid	Sensitivity (%)	Specificity (%)	AUC	*p*-value
PC(16:0/16:0)	85	95	0.953	<0.0001
PC(18:1/18:1)	80	95	0.938	<0.0001
PC(42:2)	80	90	0.915	<0.0001
PC(16:0/18:1)	95	80	0.912	<0.0001
PC(17:1/14:0)	80	90	0.910	<0.0001
PC(15:0/18:1)	80	90	0.907	<0.0001
PC(18:1e/16:0)	80	100	0.895	<0.0001
PC(16:0/16:1)	85	90	0.893	<0.0001
PC(16:0/14:0)	80	90	0.885	<0.0001
LysoPC(16:1)	80	90	0.885	<0.0001
PC(16:1/14:0)	80	90	0.88	<0.0001
PC(16:0e/16:0)	75	95	0.847	0.0002
TG(24:1/18:2/20:4)	95	65	0.845	0.0002
PC(16:0e/22:4)	65	95	0.8	0.0012
PC(18:0/16:0)	65	85	0.797	0.0013
Cer(d18:0/24:1)	50	100	0.78	0.0024
TG(22:1/18:2/18:2)+TG(18:0/18:1/22:4)	65	80	0.733	0.0119

AUC, area under the curve; Cer, ceramide; LysoPC: lysophosphatidylcholine; PC, glycerophosphocholine; TG, triacylglycerol.

### Classification of PBC lipid subtypes based on profiling

**Figure 4:**
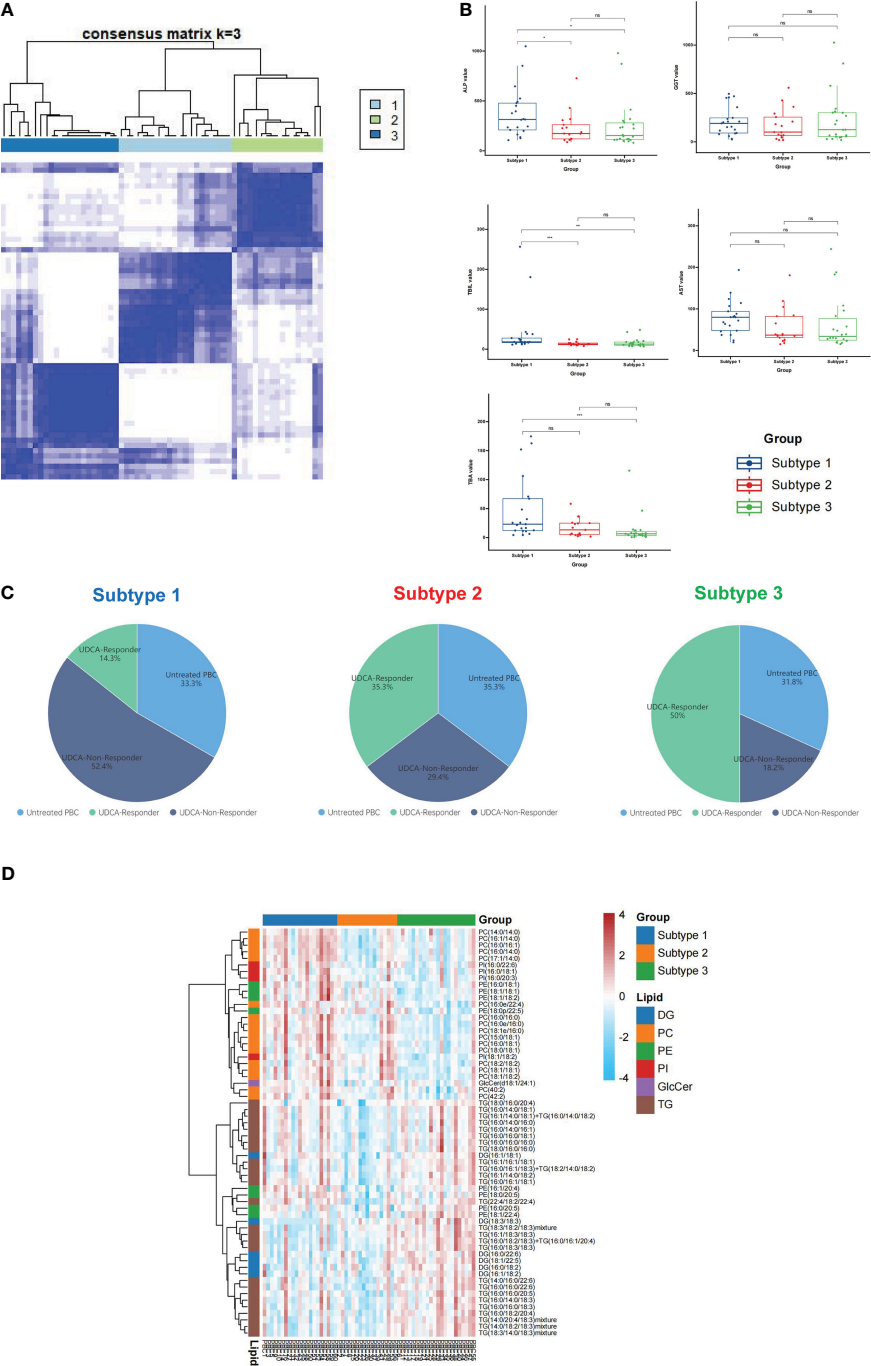
Classification of PBC lipid subtypes based on profiling. **(A)** The heatmap corresponding to the consensus matrix for three metabolite subtypes was obtained by applying consensus clustering. **(B)** PBC was classified into three subtypes based on lipid profiling; the levels of ALP, GGT, TBIL, AST, and TBA expression in three subtypes. **(C)** The distribution of untreated PBC, UDCA responder, and UDCA non-responder in subtype 1, subtype 2, and subtype 3. **(D)** The heatmap of all differential lipids related with subtype 1 (blue), subtype 2 (orange), and subtype 3 (green). DG, diacylglycerol; PC glycerophosphocholine; PE, glycerophosphoethanolamine; PI, glycerophosphoinositol; GlcCer, glycosphingolipid; TG, triacylglycerol; ns, not significant. *p < 0.05, **p < 0.01, ***p < 0.001.

A consensus matrix with *k* = 3 appeared to have the clearest cut between clusters and showed a significant association with UDCA response in patients with PBC (**Figures 4A, C**; **S3A**). In addition, the three-cluster solution was chosen as cluster number due to the relatively small incremental change in the area under the CDF curves (**Figure S3B**). This resulted in 21 PBC patients (35%) with subtype 1, 17 patients (28%) with subtype 2, and 22 patients (37%) with subtype 3 (**Figure 4A**). Combining lipid-defined PBC metabolite subtypes with laboratory examinations that reflected the disease activity and the therapeutic response, we found that ALP and TBIL were significantly higher in subtype 1 than in subtypes 2 and 3, TBA was significantly higher in subtype 1 than in subtype 3 (**Figure 4B**), and the levels of GGT and AST showed no significant alternation in the three subtypes (**Figure 4B**). In addition, we observed that the number of UDCA non-responders in subtype 1 was the highest (52.4%), whereas this proportion decreased to 18.2% in subtype 3 (**Figure 4C**).

Further analysis of the three PBC subtypes revealed differential expression of lipids, including 26 TG, 17 PC, 8 PE, 6 DG, 4 PI, and 1 GlcCer (**Figure 4D**). The heatmap showed that PC was enriched in subtype 1, but not in subtype 3. The above data indicate that subtype 1 was more severe than subtypes 2 and 3.

### Lipid profile between PBC and SS

**Figure 5:**
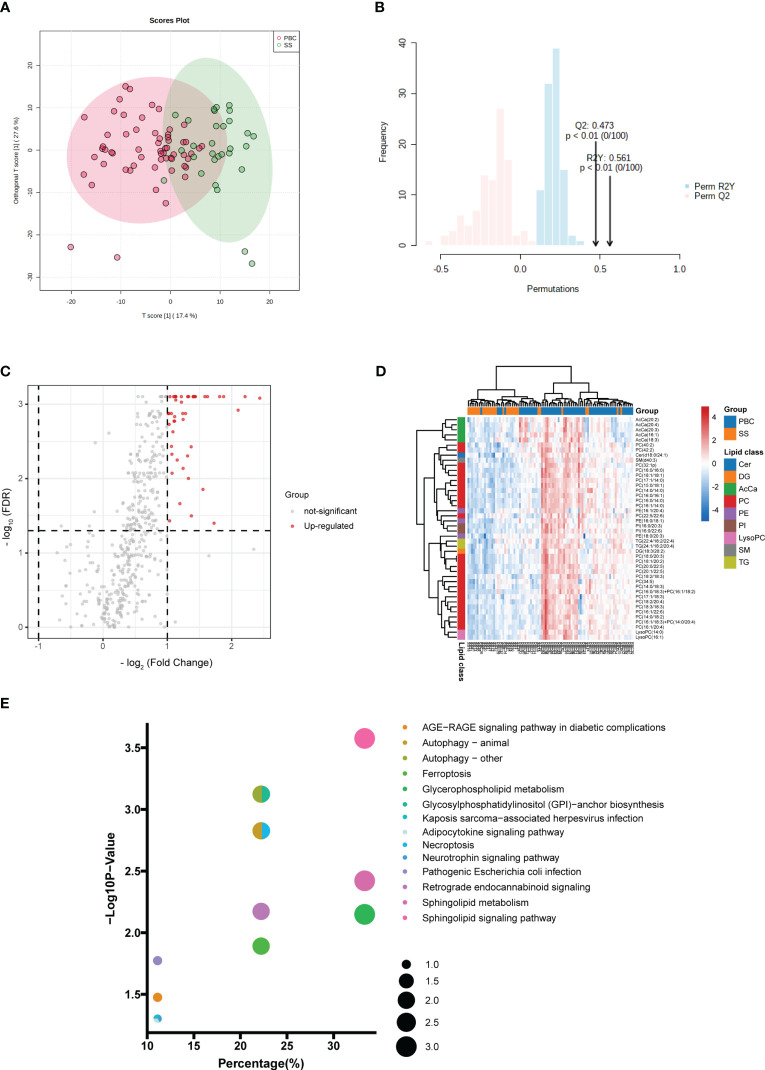
Comparison of lipid profiling in plasma PBC and SS. **(A)** Supervised OPLS-DA model shows discrimination between the PBC and SS groups. **(B)** Permutation test for the OPLS-DA model. **(C)** Volcano map of differentially expressed lipids between PBC and SS groups. Red represents the upregulated lipid and non-significant lipid molecules are gray; differential metabolic features were selected by the following criteria: FDR < 0.05 and fold change > 2.0 or < 0.5. **(D)** Clustering heatmap of the 44 lipids that were differentially expressed and VIP > 1 illustrated by the OPLS-DA model between PBC and SS groups. **(E)** Enriched metabolic pathways involving the differential lipids between PBC and SS groups. The sizes of the dots indicate the number of lipids in each pathway; the *x*-axis indicates the number of lipids in one pathway over the total number of enriched lipids, converted to percentage; the *y*-axis represents the −Log10 *p*-value. Cer, ceramide; DG, diacylglycerol; AcCa, acyl carnitine; PC glycerophosphocholine; PE, glycerophosphoethanolamine; PI, glycerophosphoinositol; LysoPC, lysophosphatidylcholines; SM, sphingomyelin; TG, triacylglycerol.

The lipid profiles of PBC and SS were investigated. No clear discrimination could be observed between the PBC and SS groups from the PCA model because of the low proportion of variance explained by PC1 and PC2 (**Figure S4**). Supervised OPLS-DA was also performed. OPLS-DA showed that samples from PBC were separated from the SS to some degree, with *R* ^2^ *Y* (cum) = 0.299 and *Q* ^2^ (cum) = 0.262 (**Figure 5A**). The permutation test was used to evaluate the overfitting of the OPLS-DA model, and the predicted residual sum of squares *Q* ^2^ (cum) was 0.473 (*p* < 0.01), and the fraction of the sum of squares *R* ^2^ *Y* was 0.561 (*p* < 0.01) at 100 permutations (**Figure 5B**). A total of 45 differential lipids were upregulated, and no differential lipids were downregulated in the PBC group compared to the SS group, as indicated by the volcano plot (**Figure 5C**; **Table S5**). Forty-four of the 45 differential lipids had VIP > 1, as illustrated by the OPLS-DA model, which was used for clustering and is shown in the heatmap annotated with lipid species (**Figure 5D**). The major differential lipid classes between PBC and SS were PC (**Figure 5D**; **S5A**) and acyl carnitine (AcCa) (**Figure 5D**; **S5B**), respectively.

### Pathway analysis of differential lipids in PBC and SS

KEGG pathway analysis was used to investigate the lipid metabolomic pathways in the PBC and SS groups. Fourteen lipid metabolomic pathways were selected and evaluated (**Figure 5E**). The differential lipids were enriched in the following nine pathways that were considered of the greatest significance [*p* < 0.05, converted lipids (percentage) > 15%]: sphingolipid signaling pathway, autophagy, GPI–anchor biosynthesis, autophagy—animal, necroptosis, sphingolipid metabolism, retrograde endocannabinoid signaling, glycerophospholipid metabolism, and ferroptosis. The detailed results of these pathways are presented in **Table S6**.

### Lipids that discriminated between PBC and SS

**Figure 6:**
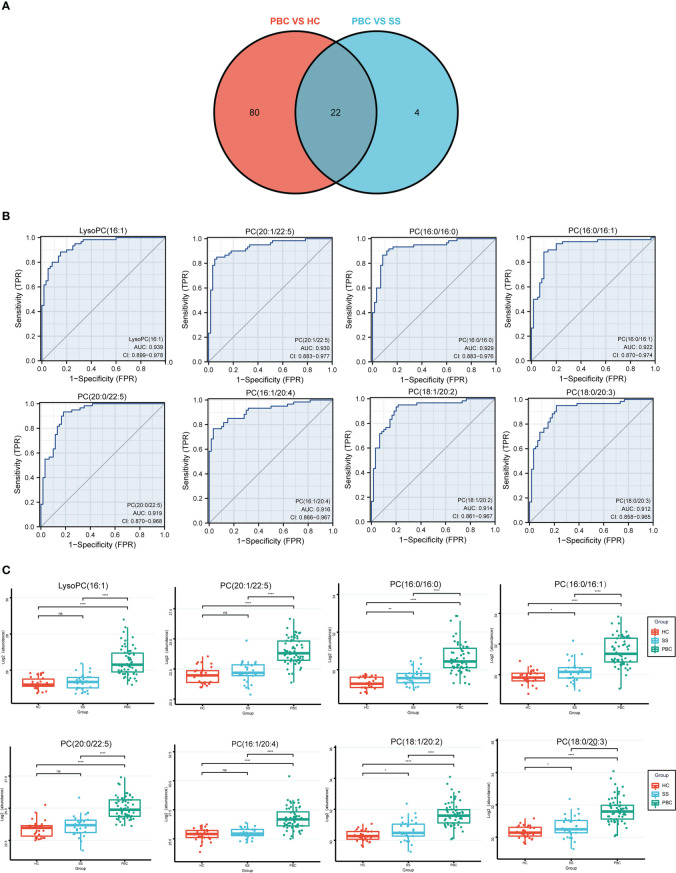
The lipids could identify the PBC and SS groups. **(A)** The Venn plot shows that 22 lipids had good diagnostic performance of PBC from SS and HC groups based on the AUC value above 0.8. **(B)** LysoPC (16:1), PC (16:0/16:0), PC (16:0/16:1), PC (16:1/20:4), PC (18:0/20:3), PC (18:1/20:2), PC (20:0/22:5), and PC (20:1/22:5) had good diagnostic performance of PBC from SS and HC groups based on ROC analysis. **(C)** The levels of LysoPC (16:1), PC (16:0/16:0), PC (16:0/16:1), PC (16:1/20:4), PC (18:0/20:3), PC (18:1/20:2), PC (20:0/22:5), and PC (20:1/22:5) expressed among HC, SS, and PBC groups. ns, not significant. *p < 0.05, **p < 0.01, ****p < 0.0001.

Further analysis was performed to detect potential lipid biomarkers for PBC diagnosis based on the differential lipids between PBC and SS, and between PBC and HC. The ROC analysis found 102 lipids with AUC values above 0.8 between PBC and HC, and 26 lipids with AUC values above 0.8 between PBC and SS. Moreover, the AUC values of 22 lipids between PBC and SS, and between PBC and HC were all greater than 0.8 (**Figure 6A**). The 22 lipids were analyzed by ROC analysis, comparing results from patients with PBC with all controls (**Table S7**). Eight of the 22 lipids were found to have AUC values above 0.9 between PBC and all control groups (**Figure 6B**), including LysoPC (16:1), PC (16:0/16:0), PC (16:0/16:1), PC (16:1/20:4), PC (18:0/20:3), PC (18:1/20:2), PC (20:0/22:5), and PC (20:1/22:5). All eight lipids were significantly higher in the PBC group than in the HC and SS groups (**Figure 6C**). In addition, PC (16:0/16:0), PC (16:0/16:1), PC (18:0/20:3), and PC (18:1/20:2) were significantly upregulated in the SS group compared to those in the HC group (**Figure 6C**).

## Discussion

Several metabolomic studies have observed significant changes in the metabolic profiles of patients with PBC. Some differential metabolites have been associated with lipid metabolism and may help diagnose PBC (12, 13, 23, 24). High levels of bile acids have been found in the plasma of patients with PBC, especially chenodeoxycholic acid, which is associated with pruritus and increases the risk of hepatocarcinogenesis in patients with PBC (25). Cholestasis, one of the characteristics of PBC, is associated with hyperlipidemia and promotes liver fibrosis, cirrhosis, and eventually liver failure (26, 27). Therefore, further investigating the association between abnormal lipid metabolism and PBC is necessary. Currently, no studies have uncovered lipid profiles in the plasma of patients with PBC via untargeted lipidomics.

Therefore, we conducted lipidomic approaches using UPLC-HRMS to quantify and identify the levels of lipid molecular species and discover new lipid biomarkers that are important to the pathogenesis of PBC. In this study, we found that 115 lipids were differentially expressed in patients with PBC compared with HC. Moreover, we revealed the association between the expression of differentiated lipids and laboratory examinations, to determine the relationship between lipids and the disease activity of PBC. Additionally, we uncovered lipids that could reflect the UDCA response in patients with PBC. Lastly, we also performed untargeted lipidome profiling compared with PBC and SS, which indicated that the lipid profile changed between the two diseases, and we evaluated lipid markers that were specific for PBC diagnosis.

Among the differential lipids that were upregulated in the plasma of patients with PBC compared with HC, TG, PE, and PC were the main differential lipid species that were enriched in PBC. TG levels increase in patients with PBC (28), and our data are consistent with these findings. Increased TG synthesis contributes to macrophage activation and forms an inflammatory phenotype in some inflammatory disorders, which also promotes the secretion of the proinflammatory cytokines IL-1β and IL-6 in macrophages (29). IL-1β and IL-6 levels are increased in patients with PBC (30). Macrophages are activated in PBC due to the elevated soluble CD163 of macrophage activation markers observed in the serum of patients with PBC (31). Macrophages are involved in liver inflammation and fibrosis in PBC (31). Therefore, macrophage activation in PBC may be associated with elevated TG synthesis. PE is one of the main components of cell membranes. The redistribution of PE across the plasma membrane bilayer is a marker of apoptosis (32). Increased apoptosis of biliary epithelial cells in PBC has been observed compared to other chronic cholestatic diseases (33), which might be associated with the elevated level of PE in this study. PC is a class of glycerophospholipids that comprises 40%–50% of biological membranes in many species. PC promotes the excretion of hydrophobic bile acids from the bile duct (34), which protects biliary epithelial cells from cytotoxic bile salts. Phosphatidylethanolamine-N-methyltransferase (PEMT) and betaine:homocysteine methyltransferase (BHMT), two PC synthesis-related genes, are upregulated in the liver of patients with PBC (35). Moreover, PC synthesis was promoted to treat the increased cholesterol and bile acid levels in patients with PBC. The enhanced level of PC in this study might be secreted from the liver of patients with PBC to reduce the injury of biliary epithelial cells to toxic bile acids.

The association between lipid levels and disease activity in PBC was uncovered in this study. Thirteen PC were positively correlated with laboratory indicators that reflect PBC disease activity. UDCA is the first-line pharmacological therapy for PBC, while PBC patients with incomplete UDCA response show a higher risk of progression to fibrosis and cirrhosis (36). We observed that six PC showed good performance in identifying UDCA responders and UDCA non-responders among UDCA-treated PBC patients. In addition, all of them were significantly higher in untreated PBC than in HC. Multidrug resistance protein 3 (MDR3) is essential for PC excretion (37) and is highly expressed in the liver of untreated PBC (38). Therefore, MDR3 may facilitate increased plasma PC levels in patients with PBC.

Furthermore, the top six UDCA response-related lipids showed a higher level in UDCA non-responders than in HC, untreated PBC, and UDCA responders. UDCA promotes the expression of MDR3 in bile ducts by activating farnesoid X receptors that regulate lipid metabolism (39). Therefore, the higher levels of PC in the plasma of patients with PBC may be associated with the upregulation of MDR3 because of UDCA treatment. In addition, we observed that PC was enriched in subtype 1 through consensus clustering analysis (**Figure 4D**). PBC patients in subtype 1 had a higher proportion of UDCA non-responders (**Figure 4C**), and higher levels of ALP, TBIL, and TBA (**Figure 4B**). ALP and TBIL are prognostic markers of PBC, and are associated with treatment efficacy, liver transplantation, and death in PBC (6, 40). TBA could also predict the liver failure of PBC (41). Therefore, PBC patients with subtype 1 had a poorer prognosis and need second-line therapy, such as fenofibrate and obeticholic acid, to reduce the risk of liver transplantation and death (40). Overall, our data showed that increased PC levels were associated with higher disease activity and UDCA treatment failure, especially for PC (16:0/16:0), PC (18:1/18:1), PC (42:2), PC (16:0/18:1), PC (17:1/14:0), and PC (15:0/18:1).

PBC and SS frequently coexist and can both be considered as autoimmune epithelitis with similar immunopathogenesis (10). This study identified differences in plasma lipid profiles when comparing patients with PBC and SS. The supervised OPLS-DA model uncovered a clear segmentation between PBC and SS patients, while the numbers of differentiated lipids were decreased in PBC and SS patients compared to those in PBC and HC. Patients with SS have abnormal lipid levels compared with xerostomic controls (42). A previous lipidomic study revealed that the species of AcCa and PC had differential expression in the serum of SS patients compared with HC (43). AcCa exert the function of transporting fatty acids into mitochondria, which are essential for β-oxidation and energy metabolism. AcCa were significantly elevated in SS when compared with PBC, while the FC of AcCa were below 2 when SS and PBC were compared with HC (**Figure S5B**), which cannot meet the criteria for a differential lipid defined in this study. All five AcCa were decreased in SS patients compared with PBC patients (**Figure S5B**), and AcCa (20:2) and AcCa (20:4) were also reduced in SS patients compared with HC (**Figure S5B**), which agrees with a study that AcCa were downregulated in patients with SS compared to HC (43). Carnitine palmitoyltransferase 1 (CPT1) contributes to the synthesis of long-chain AcCa (more than C12), whereas the CPT1 activity might be inhibited in SS patients, resulting in decreased levels of some long-chain AcCa in SS (44). In addition, AcCa (16:1), AcCa (18:3), AcCa (20:3), and AcCa (20:4) were increased in the plasma of patients with PBC compared to HC and SS (**Figure S5B**), which might reflect the impaired β-oxidation of fatty acid in PBC because of the accumulation of long-chain AcCa (45). In PBC animal model studies, long-chain AcCa increased in the liver and plasma of bile duct ligation (BDL) rats (46), and CPT1 has been observed to be upregulated in liver mitochondria of the polyI:C-induced PBC mouse model (47), which also supported our findings. Therefore, the plasma levels of AcCa showed opposite trends in SS and PBC, suggesting that β-oxidation and energy metabolism may have been altered in PBC and SS. The decreased plasma levels of AcCa in SS patients suggest that β-oxidation and energy metabolism might have been altered. PC was the main differentiated lipid class in patients with PBC compared with SS. Furthermore, seven PC showed good performance in diagnosing PBC because of an AUC of more than 0.9 when SS patients were included as disease controls in this study. Plasma levels of PC (16:0/16:0), PC (16:0/16:1), PC (18:0/20:3), and PC (18:1/20:2) significantly increased in both PBC and SS patients. In comparison to HC, higher levels of PC were found in the SS patients’ tears, saliva, and minor salivary glands (48). Therefore, altered plasma levels of PC may be a common characteristic of both PBC and SS. Further studies are needed to investigate the role of PC in these two autoimmune diseases. LysoPC (16:1) also had a good performance for differentiating PBC from SS and HC, with a higher level in patients with PBC than in the two control groups, while it did not show a significant alternation between SS and HC. LysoPC is a product of the hydrolysis of PC by phospholipase A2 (49). LysoPC (16:1) has been increased in the serum of patients with PBC with pruritus compared to those without pruritus (50). Our data showed that LysoPC (16:1) was positively correlated with TBA levels, which is a potential pruritogen in PBC. In addition, LysoPC is highly expressed in the serum of cholestatic mice (50). Therefore, LysoPC may be involved in the pathogenesis of PBC, particularly in association with pruritus in PBC.

This study had two limitations. First, we used a cross-sectional study design, so we do not know whether the observed differential lipids in the UDCA responder and UDCA non-responder groups existed before UDCA treatment in PBC. Second, the age of SS patients was younger than that of PBC patients and HC without matched age, which might have influenced our findings in the lipidomic analysis between the PBC and SS groups.

In conclusion, the differential plasma-based lipidomic signatures between PBC and HC, and between PBC and SS were revealed in this study. PC and LysoPC are specific lipid biomarkers for PBC diagnosis. Moreover, some differential PC were positively associated with PBC disease severity and could reflect the therapeutic efficacy of UDCA in PBC. Thus, disordered phospholipid metabolism may contribute to the pathogenesis of both PBC and SS. Further studies with a larger sample size from multiple centers are needed to confirm the roles of these differential PC in the pathogenesis of PBC and SS.

## Data availability statement

The lipidomics data presented in the study are deposited in the Figshare repository, https://doi.org/10.6084/m9.figshare.22590472.v1.

## Ethics statement

The studies involving human participants were reviewed and approved by the Ethics Committee of the Peking Union Medical College Hospital (JS-2156). The patients/participants provided their written informed consent to participate in this study.

## Author contributions

YLi, WL, and HL contributed to the conception and design of the study. HL, HT, SX, and YLiu collected the samples. HL, HT, LL, HY, and XM performed the experiment and statistical analysis. HL wrote the first draft of the manuscript. YLi and WL proofread the manuscript. All authors contributed to the article and approved the submitted version.

## Supplementary material

Supplementary Figure 1
**(A)** Quality control samples scattering tightly in the unsupervised PCA score plot indicated analytical performance maintained stable during analytical batch and untargeted lipidomics data is of high quality, Blue dots: QC, Black dots: samples. **(B)** Metabolite intensity RSD% distribution in QCs samples. Please see file: Supplementary Figure 1.pdf

Supplementary Figure 2 The correlation analysis of differential lipids. Please see file: Supplementary Figure 2.pdf

Supplementary Figure 3
**(A)** Consensus matrices of the 60 PBC patients from k=2 to k=6. Consensus clustering was performed on 115 differential lipids between PBC and HC. **(B)** Delta plot assessing changes in the consensus CDF area to search for ideal number of clusters. Please see file: Supplementary Figure 3.pdf

Supplementary Figure 4 Unsupervised PCA model for all PBC and SS groups. Please see file: Supplementary Figure 4.pdf

Supplementary Figure 5
**(A)** The levels of five PCs (top five PC according to the fold change from large to small) expressed among HC, PBC, and SS groups. **(B)** The levels of five AcCa expressed among HC, PBC, and SS groups. Please see file: Supplementary Figure 5.pdf
