# Supplementary material for: Plasma lipidomics of primary biliary cholangitis and its comparison with Sjögren’s syndrome
Source: Front Immunol. 2023 May 5;14:1124443. doi: 10.3389/fimmu.2023.1124443 (PMC10196160; doi:10.3389/fimmu.2023.1124443)
Supplement: Supplementary Figure 1 — (A) Quality control samples scattering tightly in the unsupervised PCA score plot indicated analytical performance maintained stable during analytical batch and untargeted lipidomics data is of high quality, Blue dots: QC, Black dots: samples. (B) Metabolite intensity RSD% distribution in QCs samples. Please see file: Supplementary Figure 1.pdf [file Image_1.pdf]

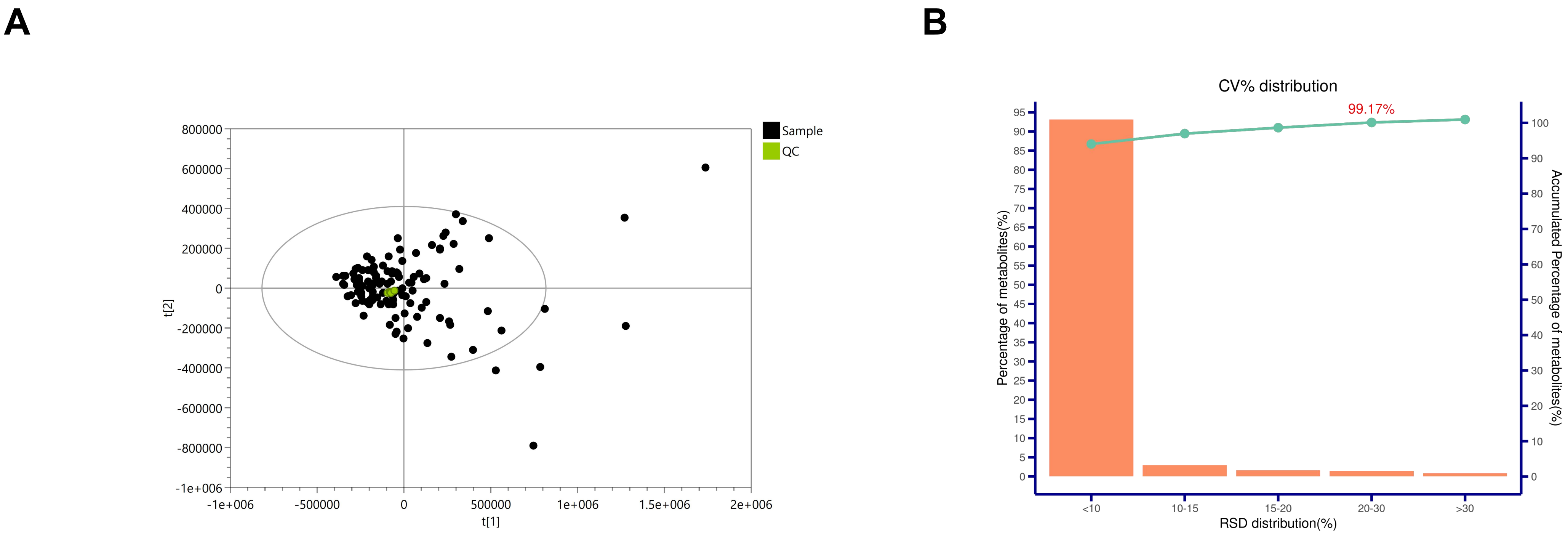

Figure S1. (A) Quality control samples scattering tightly in the unsupervised PCA score plot indicated analytical performance maintained stable during analytical batch and untargeted lipidomics data is of high quality, Blue dots: QC ,Black dots: samples. (B) Metabolite intensity RSD% distribution in QCs samples.
