# Supplementary material for: Plasma lipidomics of primary biliary cholangitis and its comparison with Sjögren’s syndrome
Source: Front Immunol. 2023 May 5;14:1124443. doi: 10.3389/fimmu.2023.1124443 (PMC10196160; doi:10.3389/fimmu.2023.1124443)
Supplement: Supplementary Figure 3 — (A) Consensus matrices of the 60 PBC patients from k=2 to k=6. Consensus clustering was performed on 115 differential lipids between PBC and HC. (B) Delta plot assessing changes in the consensus CDF area to search for ideal number of clusters. Please see file: Supplementary Figure 3.pdf [file Image_3.pdf]

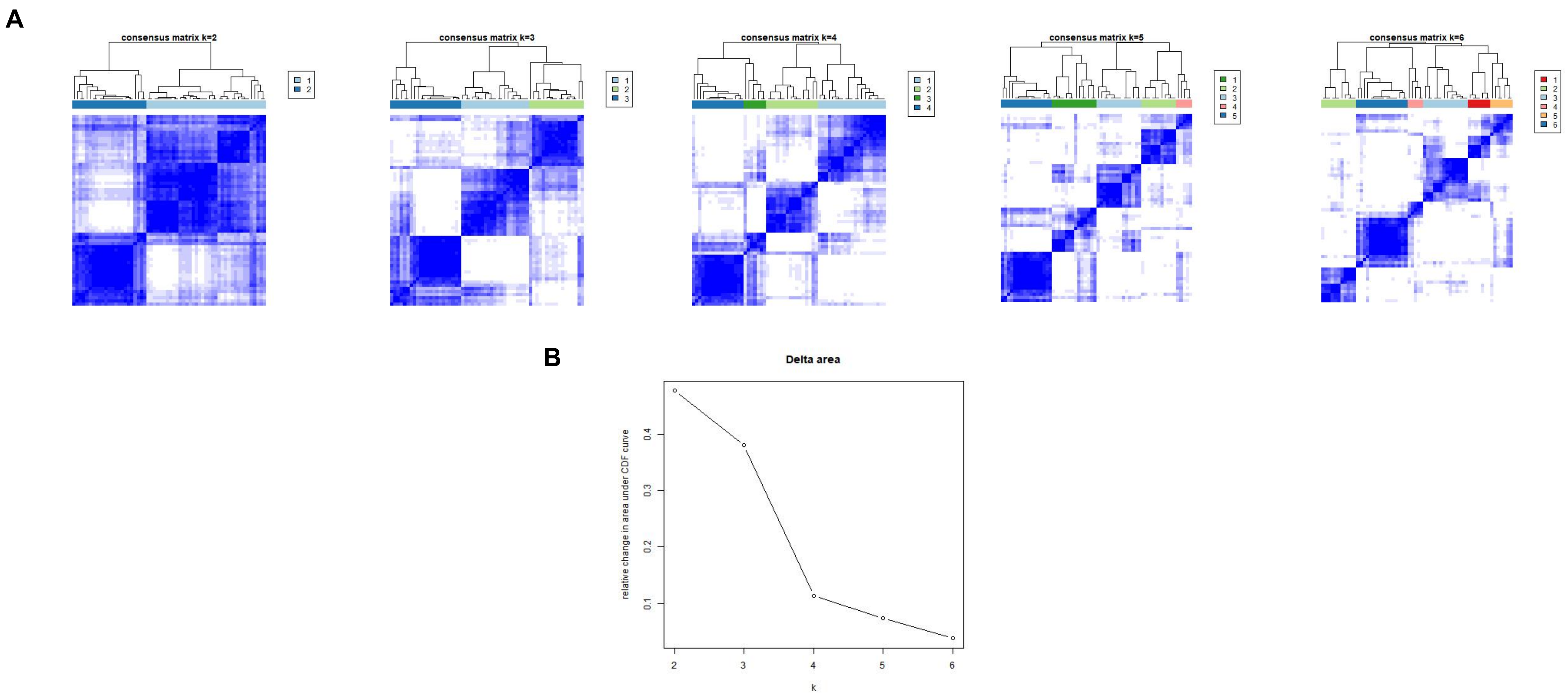

Figure S3. (A) Consensus matrices of the 60 PBC patients from k=2 to k=6. Consensus clustering was performed on 115 differential lipids between PBC and HC. (B) Delta plot assessing changes in the consensus CDF area to search for ideal number of clusters.
