# Supplementary figures and images for: Plasma lipidomics of primary biliary cholangitis and its comparison with Sjögren’s syndrome
Source: Front Immunol. 2023 May 5;14:1124443. doi: 10.3389/fimmu.2023.1124443 (PMC10196160; doi:10.3389/fimmu.2023.1124443)

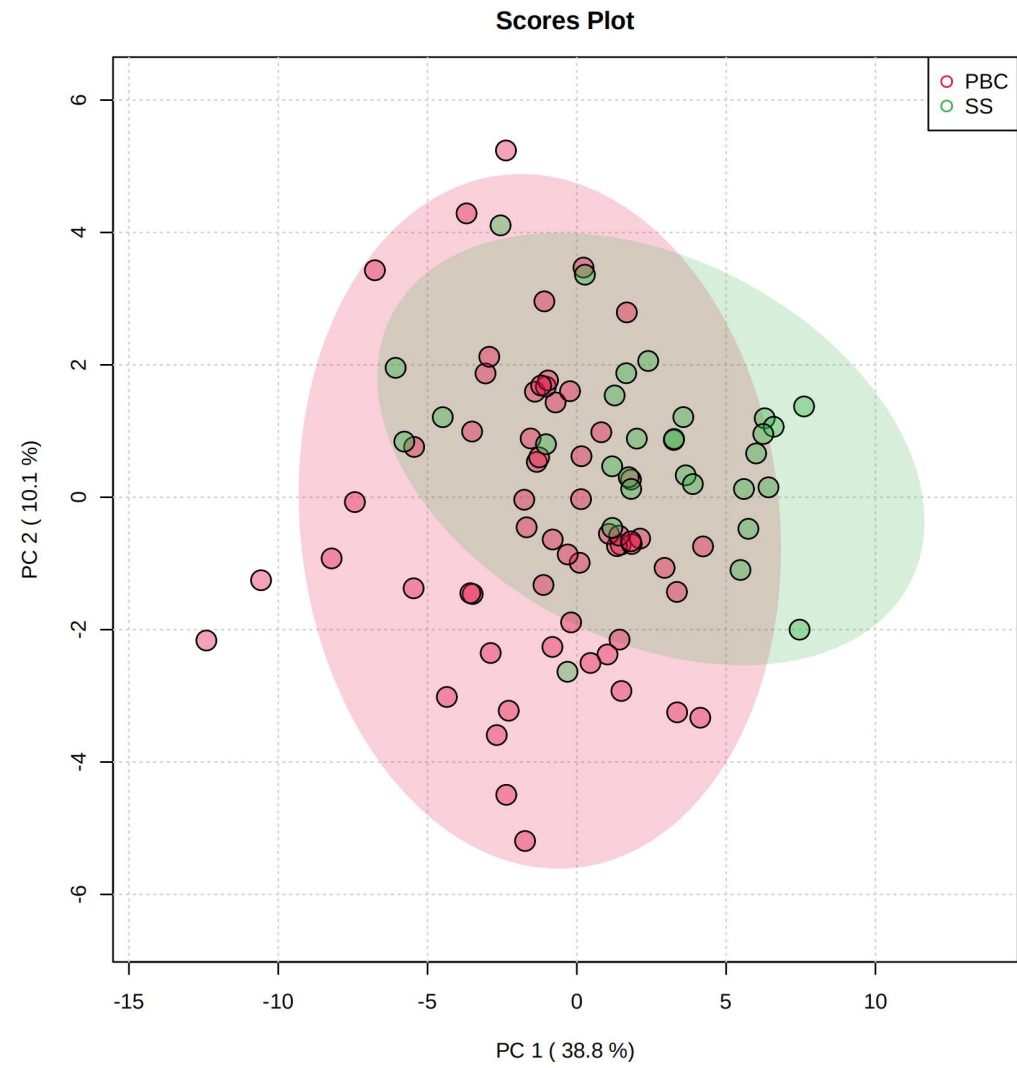

Figure S4. Unsupervised PCA model for all PBC and SS groups.

Supplement: Supplementary Figure 4 — Unsupervised PCA model for all PBC and SS groups. Please see file: Supplementary Figure 4.pdf [file Image_4.pdf]
