# Supplementary material for: Plasma lipidomics of primary biliary cholangitis and its comparison with Sjögren’s syndrome
Source: Front Immunol. 2023 May 5;14:1124443. doi: 10.3389/fimmu.2023.1124443 (PMC10196160; doi:10.3389/fimmu.2023.1124443)
Supplement: Supplementary Figure 5 — (A) The levels of five PCs (top five PC according to the fold change from large to small) expressed among HC, PBC, and SS groups. (B) The levels of five AcCa expressed among HC, PBC, and SS groups. Please see file: Supplementary Figure 5.pdf [file Image_5.pdf]

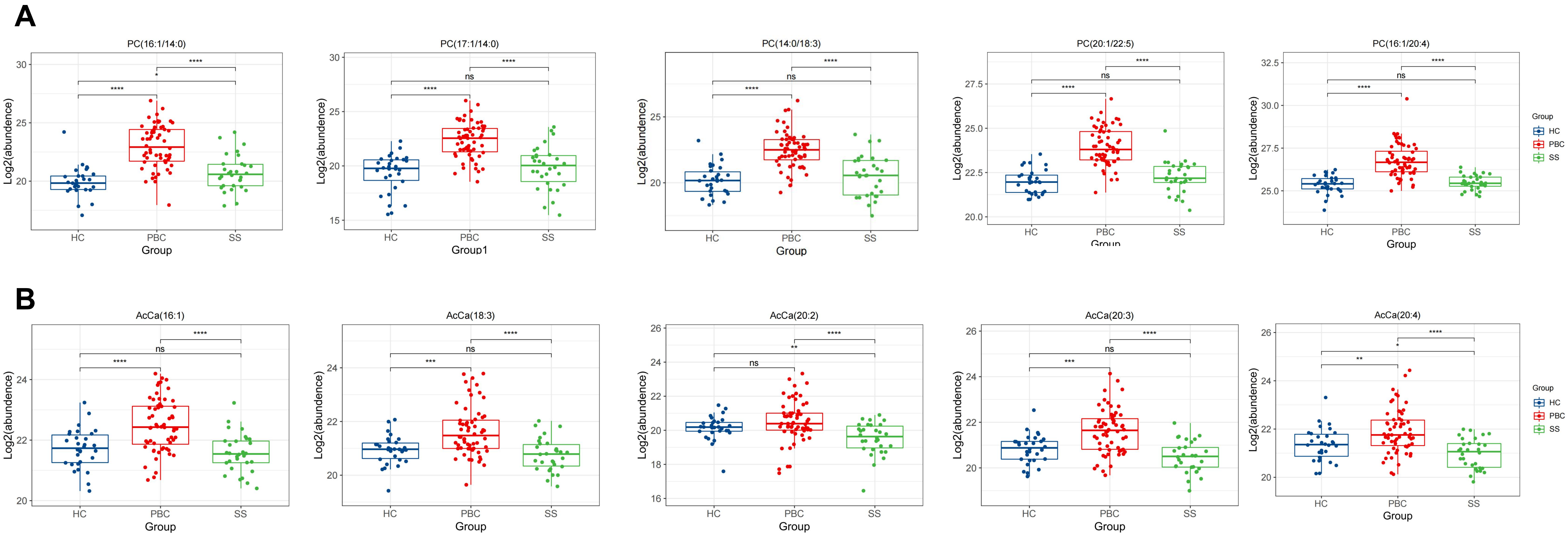

Figure S5. (A) The levels of five PCs （top five PC according to the fold change from large to small) expressed among HC, PBC, and SS groups. (B) The levels of five AcCa expressed among HC, PBC, and SS groups.
