# Supplementary material for: Plasma lipidomics of primary biliary cholangitis and its comparison with Sjögren’s syndrome
Source: Front Immunol. 2023 May 5;14:1124443. doi: 10.3389/fimmu.2023.1124443 (PMC10196160; doi:10.3389/fimmu.2023.1124443)
Supplement: Supplementary file 9 [file Table_4.docx]

Table S4. Clinical and laboratory features healthy control and three groups of PBC patients of the study.

| Group | HCs (n=30) | Untreated PBC (n=20) | UDCA-Responder (n=20) | UDCA-Non-Responder (n=20) | P-values |
| --- | --- | --- | --- | --- | --- |
| Age-year | 52.73±11.08 | 51.35±14.08 | 53.40±8.98 | 54.65±12.20 | 0.84 |
| Female-sex | 26 (86.7%) | 20 (100.0%) | 17 (85.0%) | 17 (85.0%) | 0.353 |
| ALP-U/L | 71.87±17.55 | 342.75±206.62 | 113.55±16.67 | 405.90±254.33 | ＜0.001 |
| GGT-U/L | 17.13±5.68 | 353.20±235.28 | 56.45±35.47 | 216.35±145.50 | ＜0.001 |
| ALT-U/L | 16.20±7.69 | 87.30±60.68 | 25.45±9.31 | 83.60±74.52 | ＜0.001 |
| AST-U/L | 19.17±4.49 | 92.55±49.50 | 26.15±8.06 | 82.75±54.39 | ＜0.001 |
| TP-g/l | 70.30±3.84 | 79.80±6.08 | 76.40±3.80 | 76.55±8.72 | ＜0.001 |
| ALB-g/l | 44.73±1.93 | 39.65±9.34 | 44.80±1.99 | 41.30±4.92 | 0.001 |
| TBA—µmol/l | 1.91±2.21 | 14.56±13.12 | 8.67±5.73 | 56.25±56.43 | ＜0.001 |
| TBIL—µmol/l | 12.67±3.67 | 17.04±10.81 | 13.15±3.94 | 41.32±62.52 | ＜0.001 |
| DBIL—µmol/l | 3.76±1.30 | 7.11±6.11 | 3.78±1.23 | 27.45±52.67 | ＜0.001 |
| IgG—g/l | ND | 18.09±5.36 | 13.32±1.60 | 15.11±3.86 | NA |
| IgA—g/l | ND | 2.99±1.62 | 2.64±1.16 | 2.90±1.42 | NA |
| IgM—g/l | ND | 3.88±1.51 | 3.00±1.83 | 3.22±2.70 | NA |
| ANA—% | ND | 20 (100.0%) | 19 (95.0%) | 19 (95.0%) | NA |
| AMA—% | ND | 16 (80.0%) | 16 (80.0%) | 16 (80.0%) | NA |
| AMA-M2% | ND | 16 (80.0%) | 15 (75.0%) | 16 (80.0%) | NA |
| Anti-GP210—% | ND | 3 (16.7%) ^a^ | 5 (31.3%) ^b^ | 7 (38.9%) ^c^ | NA |
| Anti-SP100—% | ND | 4 (22.2%) ^a^ | 7 (43.8%) ^b^ | 3 (16.7%) ^c^ | NA |

*Values were presented as mean ± SD. The multiple comparisons of age and serum enzymes were analyzed using one-way analysis of variance (ANOVA) and Kruskal–Wallis test (non-parametric ANOVA), respectively. The multiple comparisons of sex were analyzed using chi-square test.*

*ALB=albumin; ALP=alkaline phosphatase; ALT=alanine aminotransferase; AMA=antimitochondrial antibody; ANA=antinuclear antibody; AST=aspartate transaminase; BUN=blood urea nitrogen; Cr=Creatinine; DBIL=direct bilirubin; GGT=gammaglutamyl transferase; HDL=high-density lipoprotein; IgA=immunoglobulin A; IgG=immunoglobulin G; IgM=immunoglobulin M; LDL=low-density lipoprotein; TBA=total bile acid; TBIL=total bilirubin; TC=total cholesterol; TG=total triglycerides; TP=total protein; UA= uric acid.*

^a^ Available in 18 untreated PBC patients.

^b^ Available in 16 UDCA-Responders.

^c^ Available in 18 UDCA-Non-Responders.
