# Supplementary material for: Plasma lipidomics of primary biliary cholangitis and its comparison with Sjögren’s syndrome
Source: Front Immunol. 2023 May 5;14:1124443. doi: 10.3389/fimmu.2023.1124443 (PMC10196160; doi:10.3389/fimmu.2023.1124443)
Supplement: Supplementary file 12 [file Table_7.docx]

Table S7. Diagnostic value of the 22 selected lipid metabolites altered in PBC compared to HC.

| Lipid | Sensitivity (%) | Specificity (%) | AUC | P-value |
| --- | --- | --- | --- | --- |
| DG(18:3/20:2) | 75.0 | 85.0 | 0.855 | <0.0001 |
| PC(16:0/14:0) | 80.0 | 88.3 | 0.883 | <0.0001 |
| PC(16:1/14:0) | 80.0 | 88.3 | 0.879 | <0.0001 |
| PC(17:1/14:0) | 80.0 | 85.0 | 0.868 | <0.0001 |
| PC(16:0/16:0) | 86.7 | 91.7 | 0.929 | <0.0001 |
| PC(16:0/16:1) | 88.3 | 90.0 | 0.922 | <0.0001 |
| PC(14:0/18:2) | 73.3 | 88.3 | 0.873 | <0.0001 |
| PC(14:0/18:3) | 73.3 | 90.0 | 0.865 | <0.0001 |
| PC(15:0/18:1) | 86.7 | 80.0 | 0.876 | <0.0001 |
| PC(16:0/18:3)+PC(16:1/18:2) | 91.7 | 80.0 | 0.897 | <0.0001 |
| PC(18:1/18:1) | 68.3 | 91.7 | 0.873 | <0.0001 |
| PC(16:1/20:4) | 76.7 | 96.7 | 0.916 | <0.0001 |
| PC(18:0/20:3) | 95.0 | 78.3 | 0.912 | <0.0001 |
| PC(18:1/20:2) | 93.3 | 80.0 | 0.914 | <0.0001 |
| PC(18:2/20:4) | 68.3 | 90.0 | 0.844 | <0.0001 |
| PC(20:0/22:5) | 93.3 | 81.7 | 0.919 | <0.0001 |
| PC(20:1/22:5) | 83.3 | 95.0 | 0.930 | <0.0001 |
| PI(16:0/20:3) | 88.3 | 75.0 | 0.868 | <0.0001 |
| PI(16:0/22:6) | 68.3 | 91.7 | 0.876 | <0.0001 |
| LysoPC(14:0) | 68.3 | 90.0 | 0.846 | <0.0001 |
| LysoPC(16:1) | 88.3 | 85.0 | 0.939 | <0.0001 |
| TG(24:1/18:2/20:4) | 76.7 | 86.7 | 0.856 | <0.0001 |

Abbreviations: AUC, area under curve; DG, diacylglycerol; LPC, lysophosphatidylcholine; PC, glycerophosphocholine; PI, glycerophosphoinositol; TG, triglycerides.
