# Supplementary material for: Plasma lipidomics of primary biliary cholangitis and its comparison with Sjögren’s syndrome
Source: Front Immunol. 2023 May 5;14:1124443. doi: 10.3389/fimmu.2023.1124443 (PMC10196160; doi:10.3389/fimmu.2023.1124443)
Supplement: Supplementary Figure 2 — The correlation analysis of differential lipids. Please see file: Supplementary Figure 2.pdf [file Image_2.pdf]

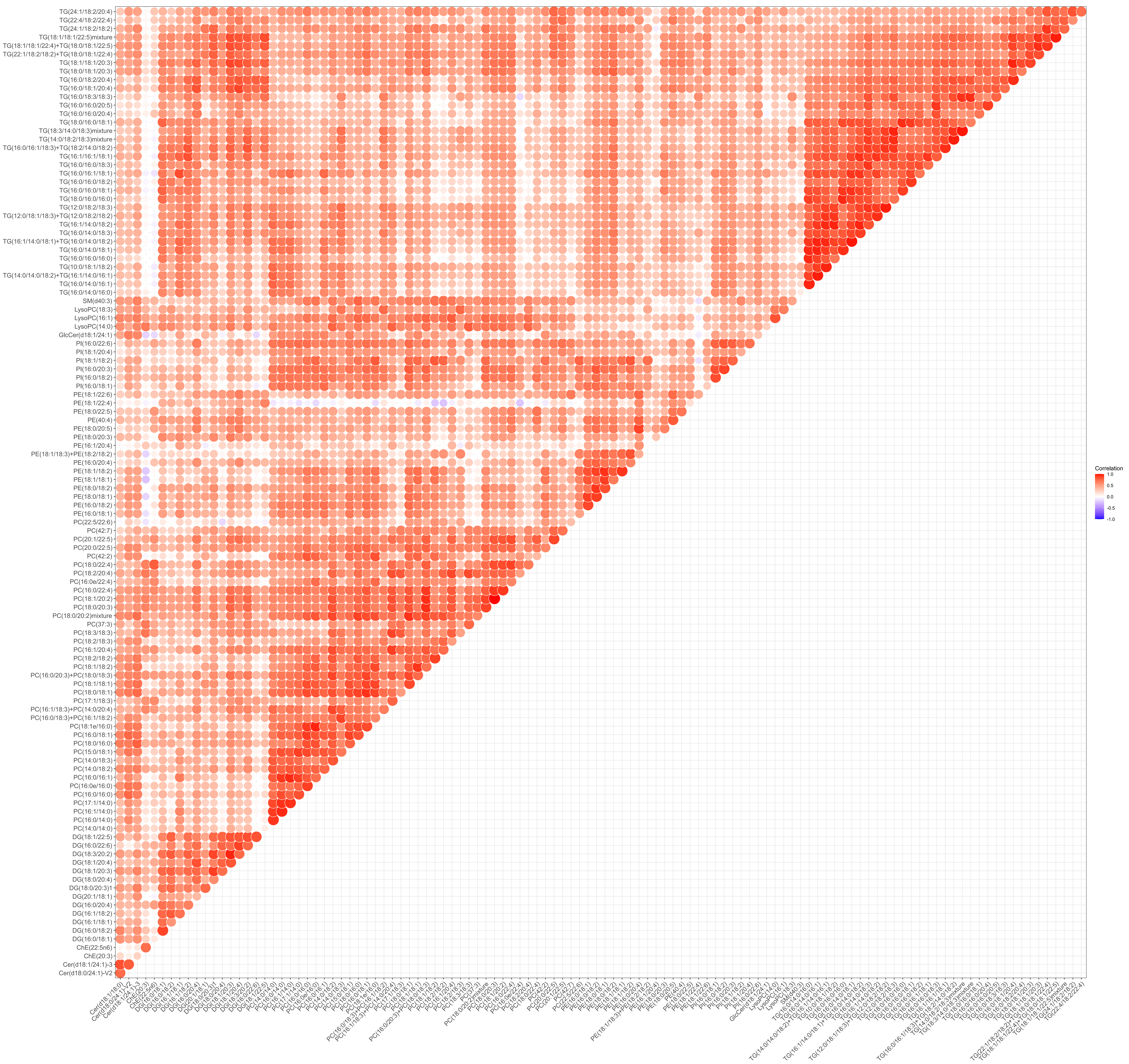

Figure S2. The correlation analysis of differential lipids.
